# A Case Study of Community—Academic Partnership in Improving the Quality of Life for Asthmatic Urban Minority Children in Low-Income Households

**DOI:** 10.3390/ijerph19159147

**Published:** 2022-07-27

**Authors:** Meirong Liu, Jae Eun Chung, Jiang Li, Brianna Robinson, Florencia Gonzalez

**Affiliations:** School of Social Work, Howard University, Washington, DC 20059, USA; jaeeun.chung@howard.edu (J.E.C.); jli@howard.edu (J.L.); brianna.robinson@bison.howard.edu (B.R.); florencia.gonzalez@howard.edu (F.G.)

**Keywords:** community–academic partnerships, community-based participatory research, nonprofit organization

## Abstract

Community–academic partnerships (CAPs) are being increasingly used to study and address health disparity issues. CAPs help to create new bodies of knowledge and innovative solutions to community problems, which benefits the community and academia. Supported by a grant, a partnership was formed between an academic research team and a community health organization to analyze and interpret data collected from the caregivers of asthmatic African American children living in urban low-income households. Using a case study approach, we discuss how we built a healthy CAP and the lessons learned from the process. Our analysis was guided by the six main factors that facilitate success in developing collaborative relationships, including (1) environment; (2) membership; (3) process and structure; (4) communication; (5) purpose; and (6) resources. Based on these six factors, we describe our collaboration process, challenges, and areas for improvement. We aimed to provide a “points-to-consider” roadmap for academic and community partners to establish and maintain a mutually beneficial and satisfactory relationship. Collaborating with community members and organizations provides unique opportunities for researchers and students to apply their skills and knowledge from textbooks and the classroom, engage with community members, and improve real-life community needs. Building a constructive CAP involves efforts, energy, and resources from both parties. The six major themes derived from our project offer suggestions for building a healthy, collaborative, and productive relationship that best serves communities in the future.

## 1. Introduction

Research on improving community health and reducing health disparities has often been carried out in one direction, in which academic researchers conceptualize and implement research projects with minimal input from community stakeholders [1]. The dissemination of knowledge and information has also targeted the academic audience rather than community members [2]. Such research often fails to address the most critical needs of the community. These findings could not be translated from university-based to “real world” settings [3]. Community–academic partnerships (CAPs) create unique opportunities to draw the respective strengths and expertise of academic researchers and community stakeholders to address these challenges.

Under CAPs, communities and researchers in academic institutions form partnerships in conducting research to increase research relevance and feasibility [1]. On the one hand, community stakeholders provide firsthand knowledge and insight that could help to identify critical public health concerns and to design and implement research projects [4]. On the other hand, involving community stakeholders also helps decrease the marginalization of communities that have historically received little benefit from research participation [5]. In addition, collaborating with community members and organizations provides unique opportunities for researchers and students to apply their skills and knowledge from textbooks and the classroom, engage with community members, and address real-life community needs. Further, it “increases services to the community, access to grants, access to populations for research, access to university library resources”, among other benefits that are specific to organizations, communities, and academia [6], p. 404).

Community-based participatory research (CBPR) is one of the most widely discussed paradigms for promoting health equities in communities and providing guidance for CAPs [7,8]. As an implementation approach, CBPR emphasizes equal involvement of community members, organizational representatives, public health practitioners, and researchers throughout the research process. All partners contribute expertise, share value, and integrate knowledge with interventions and policy advocacy to improve quality of life for marginalized communities [9]. CBPR requires researchers to cultivate cultural humility, not assuming one’s own identity, culture, and perspectives are superior to those of others and being interested in the identity, culture, and perspectives of others [10]. It motivates researchers to recognize and critically examine how their own intersecting social identities, such as race, gender, ethnicity, and socioeconomic status, can impact on their own and the community’s engagement in research [10]. In CBPR, active collaboration of diverse stakeholders is prioritized to provide marginalized communities an opportunity to voice their concerns, weigh in on potential solutions, and address social justice issues that matter to them [11]. Community partners have equal authority and responsibility with the academic research partners. In addition, the CBPR partners negotiate roles and responsibilities depending on different expertise brought by members of the community and of the academic research team as well as the different goals and motivations of each party, both before the research begins and throughout the research process to ensure that the concerns, interests, and needs of each party are addressed [12]. Flexibility, mutual respect, recalibration, and a profound commitment to the projects’ aims enable the CAPs to move forward and succeed [13]. Ref. [14] described a CBPR conceptual model that contains four domains: (1) external and internal contexts (i.e., social-economic factors, capacity of stakeholders, and history of trust or mistrust); (2) structural (i.e., diversity, shared resources, and agreements) and relational (i.e., decision making, respect, dialogue) elements of partnering processes; (3) intervention and research design that are co-created with community and cultural knowledge; and (4) intermediate outcomes (i.e., empowerment, shared power in research, and project sustainability) and long-term outcomes (such as improved health and health equity). Systematic reviews and individual studies have increasingly documented the effectiveness of the CBPR approach in supporting and empowering community, building sustained partnerships, and promoting healthier behaviors and policy changes, as well as improving health outcomes [15]. Ref. [14] also summarized CBPR’s strategies, including integrating community-based practice and knowledge into evidence-based practices [16]; addressing the inequitable power differentials between researchers and community partners [17]; and seeking policy and system-change outcomes toward social justice [18]. Researchers have also identified the elements of CBPR that are associated with positive outcomes, including community partnership participation in (1) a study advisory committee; (2) data collection; (3) development of interventions; and (4) participant recruitment [19].

## 2. Objectives

The current article describes a case study of the Child Asthma Study Partnership and its contributions to improving the quality of life for asthmatic African American children living in urban low-income households. This case study highlights the process of building a partnership between the team of interdisciplinary researchers and a community-based non-profit health organization. The goal of the partnership was to address challenges faced by the non-profit community health organization in managing and analyzing data for its organizational goals and to enhance the usability of data for the health of asthmatic children in low-income families. We aim to discuss the process of developing rapport and partnership and the lessons learned from the process.

## 3. Methods

The interdisciplinary research team at one of the Historical Black Colleges and Universities (HBCUs) received a National Science Foundation (NSF) grant to conduct research addressing community health organizations’ needs in analyzing and interpreting health data and to provide data analysis training for students. Our project aimed to develop a Child Asthma Partnership as an approach to support a community organization’s data management and analysis needs and to enhance the usability of data for the health of asthmatic, urban, and minority children in low-income households. Those who were involved in the Child Asthma Partnership included university researchers and community non-profit organization staff. The outcomes from the project are discussed in another paper (author, under review) and the current case study is to discuss the process of collaboration. The process of building a constructive and collaborative partnership is not automatic, as it involves effort, energy, and resources from both parties. Using a case study approach, we discuss how we constructed a healthy partnership and dealt with challenges we encountered, and share lessons learned from the partnership process.

The community organization involved in the described study is a non-profit health organization providing asthma health programs for low-income African American households in the District of Columbia (DC), especially for Ward 8. Ward 8 is home to DC’s most underserved neighborhoods for health care with 92% of residents being African American and having the lowest median household income in DC. The staff members of the community organization are practitioners who provide health education and intervention. Located in Ward 8, the community organization works directly with community residents and leaders through a series of evidence-based community health interventions, health education, community partnerships, and policy advocating activities. It represents the voice of residents with lung disease issues in underserved communities with the goal of improving of their lung health. The staff on the academic team are university academics with interdisciplinary backgrounds and research experiences on health disparities.

The distinctive characteristics of the community health organization and the academic team can influence the power dynamics. For example, the definition of the data collection by the community health organization may not fit the academic objectives; on the other hand, a standard definition of the data collection by the academics may ignore some realities of the underserved communities’ experience (better known to the community health organization). In addition, in terms of methodologies, there is an asymmetry between the community health organization and academia in their understanding of techniques to analyze and interpret the data. Further, in terms of objectives, the academic team may prioritize the visibility of their research, while the community health organization aims for actionable changes to improve the life of communities. There is no alignment between the criteria for publishable, successful research and those for clear, simple messages for policymakers.

To overcome those asymmetries and possible divergences in objectives, the academic team should aim at (a) recognizing the superior experience of the community health organization in grasping realities faced by underserved racial minority communities, (b) educating the community health organization on the basics of their methodologies (e.g., the need for unbiased interpretation of data), (c) transparently acknowledging their publication objectives for their research, and (d) delivering, to the extent possible, actionable messages in non-technical terms for the community health organization to use in their service for disadvantaged communities and interactions with policymakers and other stakeholders.

In this study, an unstructured participatory observation method where the researcher is also a member of the group being observed [20] was used to describe the process of developing a CAP through an analysis of interactions that occurred between the team of interdisciplinary researchers and the community health organization. Sources of data collected during this 2.5-year project period included observation of archival records, such as meeting records, and direct observation of study participants. Each team member produced a list of challenges and points for improvement, had a discussion, and summarized the findings. Observation methods were used to examine professional practices and communication between health professionals, such as team functioning and communication [21]. Such methods allow the researcher to actually see what people do rather than what they say they do [22]. Systematically observing people in naturally occurring contexts can reveal much more information than what individuals may recall or choose to report in comparison with other self-report data collection methods [21]; such methods have been increasingly applied in health sciences research [23].

Our analysis was guided by the six main factors that facilitate success in collaborative relationships, as explained by [24]: (1) environment (e.g., history of collaboration), (2) membership (e.g., mutual respect), (3) process and structure (e.g., flexibility and clear roles), (4) communication (e.g., open and frequent communication), (5) purpose (e.g., concrete, attainable goals and shared vision), and (6) resources (e.g., sufficient funds and skilled convener). Based on these six factors, we describe the process of our collaboration, the nature of our partnership, challenges, and areas of improvement. We also follow the CBPR paradigm for its focus on relationships between academic and community partners, with the principles of co-learning, mutual benefit, and long-term commitment and incorporation of community theories, participation, and practices into research efforts [8].

## 4. Results

### 4.1. Environment

Environmental factors include the geographic location and social context within which a collaborative relationship exists [24]. In order to form the Child Asthma Study Partnership, the research team reached out to a community liaison who has expertise in CBPR approach and has played a critical role in connecting researchers and community organizations. The liaison asked the community health organization its interest in working with university researchers and introduced the researchers to the community health organization via email. Through email communication, the researchers and members of the community health organization set a time to meet at the organization’s office. During the first meeting, the researchers explained the nature of the project and made formal introductions. The meeting served as an opportunity to understand each other’s needs and resources and find an avenue for potential collaboration. The researchers and the community health organization agreed to work on the data from the organization’s intervention programs for asthmatic youth in low-income communities. After receiving the de-identified data from the community health organization, researchers worked together with students to analyze and interpret the data. The data were transformed into meaningful figures to inform how the community health organization can best serve the communities, attract potential funders, and achieve their mission of improving lung health of low-income communities.

The initial step of forming the Child Asthma Study Partnership was facilitated and eased because the liaison had sustained an existing rapport with the community health organization. The liaison participated in the first few meetings between researchers and the community health organization members until there was a firm understanding of project goals and mutual understanding of each other. After a few months of communication via face-to-face meetings, emails, and phone calls, researchers and the community health organization members developed a history of collaboration with the community organization and then further established a sense of trust and understanding with the community over the goals of conducting research.

Although the two parties did not have a history of working together, the commitment of both parties to advancing health equities for African Americans facilitated the communication at the beginning. The university where the researchers belong has a historical mission of serving the underserved; and the community health organization is located in Ward 8 where the neighboring communities are comprised majorly of African Americans in dire need of better health service and access.

From our experience of team building, we learned that team building takes time and resources and can be facilitated by involving a community liaison. Having a community liaison is beneficial in searching for and developing a collaborative partnership with community organization. A community liaison also helps to sustain mutually rewarding, productive, and culturally appropriate collaborations between academic institutions and community organizations [25]. However, although this project was funded by a grant, our budget did not include enough resources for team building. We were not able to compensate for the times and efforts the community health organization members spent for the project, and that was difficult as we competed for already limited resources the community health organization had. For any researchers and organizations looking for building a CAP, we recommend planning in advance and including a budget for partnership development. It is important to note that some grants are made specifically for team building. For example, the Patient-Centered Outcomes Research Institute (PCORI) has a funding mechanism specifically for team building. Its stakeholder convening support grant provides a budget to hold multi-stakeholder convenings, meetings, and conferences and to bring together diverse stakeholders around an issue of common health interest. 

### 4.2. Membership Characteristics

Membership characteristics refer to the skills, attitudes, and opinions of the individuals in a collaborative group and the culture of the organizations which form the collaborative group [24]. From our partnership, we learned that it is critical to consider establishing a representative from both the non-profit organization and academic research team to be present at all meetings throughout the research study. The representative would be knowledgeable of their teams’ respective goals and objectives. Without one present, challenges can quickly arise when it comes to interpreting data. For example, one of the difficulties that we experienced at the early stage of the collaborative project was understanding and interpreting the data, such as questionnaire design and the process of data collection. Therefore, being able to easily reach and contact the organization supplying the data is essential when one party lacks knowledge of survey design and the data collection process. Having a representative from the community health organization who can communicate directly with academic researchers not only helps receive timely feedback and have answers about the unknown questions but ensures that the academic team is interpreting the data to fit the needs of the community health organization, e.g., what the community health organization hopes to learn from the data. For example, in our work on assessing the perception of asthma triggers with asthmatic children’s caregivers, the community health organization’s representative was able to recommend what kind of child asthma triggers were most important in preventing pediatric asthma. Based on such communications, the research team was able to focus on the data analysis that provided such information to identify resources needed for the organization’s effort to reduce asthma triggers for African America families in low-income households. Such knowledge transfer and exchange help community organizations more effectively translate research findings into practical interventions. Previous studies also suggested several steps for effective knowledge transfer, including identifying the types of messages to be transferred and where they should be drawn from (e.g., empirical studies and systemic reviews), the target audience to ensure the messages from research are presented in a way that is meaningful to them, and credible messengers who may have greater access to or influence among target audiences [26].

Coalition building plays a critical role in successful CAPs and in promoting the use of evidence by policymakers and practitioners. The key dimensions of coalition building include pursuing mutual benefits, building trust, clarifying the roles, and managing conflict [27]. Successful CAPs are found to pursue mutual benefits, such as a specific agreement that ensures strategic advantages for both parties, smoother facilitation of contracts, financial incentives for the university, actionable research, innovative ideas, improvement in the quality of services, and facilitation of knowledge translation to direct practice [28]. Trust plays a key role in the sustainability of partnerships, leading to continued work, additional projects, and system-level changes. Clear delineation of roles among partners related to developing research questions and methodology, as well as the eventual dissemination of the findings, is essential to CAPs [29]. Further, effective conflict management skills are vital in building CAPs that lead to using research evidence to inform practice.

Another lesson we learned from our process of CAP is the need for a balance and understanding from researchers to wait and consider the other obligations and responsibilities of the other parties involved. Since the agendas of researchers may not always align with that of the NPO, meetings must be conducted in a manner that is direct and focused. Questions and concerns should be written up prior to any meeting, and notes should be taken during meetings for future reference. Notes should include any new information, answers to questions, and a list of tasks to complete for the next meeting. Not only does this promote respect and understanding but it also helps ensure collaboration success. Utilizing time effectively in this manner is crucial for success. Arriving at the meetings prepared with questions also demonstrated the commitment to the research questions that the community health organization identified.

Our study embodied membership by including a representative from each group in the decision-making process after data analysis. This inclusion was not limited to decisions surrounding the original study of interest, such as presenting data findings and information included in the written report. It also extended to invitations to work in collaboration on future projects. We also encouraged membership by inviting other interested parties from the community health organization to join the meetings to see if they had any suggestions or feedback to contribute to the findings.

### 4.3. Process and Structure

Process and structure refers to the management, decision making, and operational system of a collaborative effort [24]. Throughout this study, we ensured that the community health organization had a stake in all processes surrounding the data results. We also involved the organization in the process of determining authorship as it relates to publishing findings. We shared our written work before submission with the organization for them to review, make suggestions, and add any relevant information. For example, revisions by the organization were made to include the organization’s mission statement and add additional background information that made the results more meaningful and helpful in securing additional resources for the community being served. This promoted a shared sense of ownership in the decision-making process and made certain that pertinent information about the organization was included.

Having a flexible schedule is also essential to the operation and management of working with community organizations. Considerations when scheduling meetings include accounting for differences in time zones and suggesting multiple dates and times to fit the schedule of all team members. This can be accomplished by finding times that work best for the research team, then proposing those times to meet with the organization. In this partnership, we had a relatively small research team and were able to find multiple dates and times that work for team members. These dates and times were then proposed to the community organizations to fit into their schedule. We understood the importance of having each stakeholder present during meetings, ensuring that everyone could participate in the decision-making process.

### 4.4. Communication

Communication refers to the channels used by collaborative partners to send and receive information, keep one another informed, and convey opinions to influence the group’s actions [24]. Researchers need to be transparent about the extent of community participation as well as more thoroughly and accurately describe the nature of the partnership with members of minority communities in order to build upon the scientific literature on community-engaged methods [19]. In this study, open and transparent communication was maintained among the research team through weekly meetings and emails with all team members included. Without having open communication channels with the community organization, interpreting the data in a useful way was challenging. Future research partnerships should consider spending sufficient time on every meeting to gain an understanding of every member’s role and the significance of the project from each group’s perspective. Thus, having a member of the community organization involved in these discussions with the principal investigator and other academic professionals on the research team would be helpful in bridging the gap and adding more meaning to the data and results. For example, the researchers did not always recognize how some of the questions were designed on a survey questionnaire by the community health organization on asthma reduction. Specifically, there was initial confusion on the significance of knowing if the child slept in their own bed or with the caretaker and if there was a water leak in the households. During one of our follow-up sessions, we learned that information about the child’s sleeping arrangement was necessary for the organization to provide the correct pillow and mattress cover to the asthmatic children, and that water leaks could increase the chances of mold being present in the households. If such questions were never discussed with the organization, then time would have been spent on interpreting other aspects of the data that may not be as significant to the organization as the data that appeared irrelevant. Without open communication and both parties being equally involved throughout the process, the data analysis and interpretation can easily become divided or misguided. 

In addition, the academic institution and partnering community organization should establish appropriate means of communication at the beginning of any research study. Although email is still preferred in many professional realms, the convenience of calling or sending a text message makes communication much more effortless when questions arise from understanding survey instruments and data collection procedures. In our partnership, we relied primarily on email as the form of communication, although we would occasionally call and send text messages to contact. We found that this ensured efficient and open communication.

Despite the need for improving communications in collaborative partnerships, these challenges can be overcome by ensuring a representative from each party is present at all meetings. By doing this, the goals of each group can be at the center of the research study. Furthermore, establishing preferred communication methods and setting standards and expectations about the turnaround time is vital for maintaining deadlines and building solid relationships. Overall, the trust and relationship between academic institutions and community organizations should continue to be built through collaborations such as assisting the organizations in analyzing data and interpreting findings to best inform organizations’ efforts in community services. Through these collaborative projects, both academia and the community organization can continue to mutually support each other’s efforts to improve community health.

The study took place from Fall 2019 to Spring 2022, and the outbreak of the COVID-19 pandemic had an impact on the communication. Due to the lockdown and various of safety measures from March 2020, communication shifted from in-person meetings and activities to virtual meetings and activities. In our study, some levels of communication were conducted informally such as by regular email updates. However, in-person meetings provide opportunities for partners and team members to informally get to know one another and build trust and strengthen their relationships. Spending informal time together as a group over meal (e.g., potluck) would be a fun activity and would an excellent way for partners to further bond with each other.

In addition, we benefited from the National Institutes of Health’s Clinical and Translational Science Award (CTSA), in particular, the Georgetown-Howard Universities Center for Clinical and Translational Science program. This program provided an opportunity of connecting the academic team to community liaison who had built connections and had experiences of working with community-based organizations. Mutual understanding was reached after introducing and communicating the backgrounds of the researchers and the nature and objectives of the grant, as well as the backgrounds, objectives, and needs of the community health organization. The liaison served as a facilitator and was critical in building trust, addressing the needs of, and ensuring mutual benefit for the partners. Since the goal of the partnership was to address challenges faced by the community health organization in handling data for health promotion needs and to enhance the usability of data for the health of asthmatic children in low-income families, we spent time gaining a thorough understanding of the community health organization’s mission, programs, and challenges. We then aligned academic needs based on the community organization’s needs in analyzing and understanding asthma data to inform their practice on asthma reduction for underserved African American families. Based on the community health organization’s needs of assessing asthma triggers, the academic team formulated preliminary research questions. The preliminary research questions were then sent to the community health organization for feedback and to see if there are modifications or additional research questions. 

### 4.5. Purpose

Purpose refers to the reasons for the result or vision the collaborative group seeks and the specific tasks or projects the collaborative group defines as necessary [24]. One of the challenges at the beginning of analyzing the community health organization’s data was understanding the purpose or vision of what the organization hoped to accomplish from the project. Since we were unsure about the significance or meaning of specific data, such as the list of several differently labeled follow-up dates, deadlines were pushed back to accommodate the time needed to schedule meetings with the partnering organization and conduct follow-ups. In addition, the research team had challenges in generating sufficient research questions at the beginning of the project because we did not know what the organization had hoped to achieve or learn from analyzing the data. Research questions were added throughout the project, mainly when we presented the first draft of the data report to the organization. The graphs and charts prompted more questions that changed the focus of the research questions. For example, as the organization staff gained more understanding of the data after we presented the initial findings, they became interested in comparing the results based on whether the participant lived in a multi-dwelling or single-family home, which has important implications for addressing child asthma triggers. Therefore, we analyzed the data further to reflect the new research question and examined factors such as the experience of water leaks and comparison of pest management referrals with mice and roaches as child asthma triggers, as well as the last time furnace filters were changed by the type of homes.

Despite this initial setback, we attained a mutual understanding of the goals of this collaboration and adjusted how we approached the data. Although we had different approaches to the purposes of the project, we were able to align the project to fit the needs of both the community and academia. While finding the answers to the questions proposed by the organization and suggesting improvements in their questionnaire design, the research team used the data findings and collaborative process to develop additional relevant research studies and publish results in academic journals.

Moreover, a shared vision was quickly developed as the project continued and led to building and maintaining relationships in future projects. This shared vision included the recognition of using data to help secure additional funding to provide essential resources to impacted communities, determining which trigger factors relating to asthma were most relevant to the participants compared to the United States, and using the results to improve the effectiveness of future projects.

### 4.6. Resource

By securing funding through the NSF grant at the beginning of the project, the research team utilized those financial resources to hire a graduate student to analyze the data. By using software made available to faculty and students at the university, data were analyzed with the Statistical Package for the Social Sciences (SPSS) at no additional cost. This allowed the team to delegate tasks and manage time more efficiently to meet scheduled deadlines.

Although the time of the academic team members and graduate students was financially compensated by the grant, our budget did not include the fund to cover the time NPO staff spent on the project.

Outside of financial resources, human resources, such as conveners, can also increase the success of partnerships. For this project, the principal investigator (PI) would be recognized as the convener, although this role was not exerted. Instead, this resource was not necessarily important given the small size of the team and the amount of effort from both sides to contribute equally to the project.

## 5. Conclusions

This descriptive case study discusses the process of collaboration between a team of university researchers and a community health organization in analyzing and utilizing data to examine asthma triggers for asthmatic children in low-income households for better service delivery and resource allocation. The six major lessons derived from the Child Asthma Study Partnership offer suggestions for future CAPs on building a healthy, collaborative, and productive relationship to establish and achieve mutually satisfying goals. We summarize the challenges and lessons we learned from the Child Asthma Study Partnership in Table 1, which we hope can provide points for consideration for any researchers and community organization trying to build a team and collaborate.

CAP is more relevant than ever, especially within the domain of health [1,30]. The increasing health disparities call for partnership between academia and communities [31]. The two parties need to collaborate to find a solution as they often bring unique expertise and connections to the table. Particularly, among African American communities, the distrust and mistrust are deep in roots and the engagement of community-embedded organizations is essential to generate the involvement of communities. For this project about pediatric asthma, the academic researchers would not have been able to conduct the survey and visit the families with asthmatic children on their own as they lack connections to these families. One needs the trust to visit these families and conduct surveys and home inspections. The community health organization had those trust and connections and yet they did not have enough manpower and resources to analyze the data to its full potential. The researchers from academic institutions, through collaboration with community health organization staff, were able to parse out meanings. 

As we described in this paper, the process of CAP was not free from challenges as the two are operating and running with different goals and interests. Throughout the collaboration process, we had to understand that the two parties work with audiences and stakeholders of different nature and have unique goals and performance evaluation criteria. For example, academic researchers are interested in publishing their findings in journals. The community health organization has needs to appeal to potential funders to sustain its operation, and thus, the results from the analysis need to be appealing to the eyes of the potential funders. What was necessary for our CAP was regular communication to find common ground and conversation to align each party’s unique needs with the project’s goals and aims. At the same time, we also realized that excessive time commitment and unclear expectations regarding roles and responsibilities from both parties could be a hindrance. 

In our case, the CAP was limited to the data analysis stage only, although the CAP can be applied to the complete aspects of the research activities, including planning, development, design, implementation, analyses, and dissemination of research findings. Still, the lessons we learned from the CAP are relevant to any team which involves members from both academia and communities. Some key lessons we learned from the CAP include (1) the need for clear, open, regular, and transparent communication between partners; (2) building of mutual trust and respect for differences in foci, work nature, expectations, and cultures; (3) the importance of establishing a shared vision (e.g., positive community impact, dedication to serving the underserved neighboring communities, etc.) and sense of co-ownership and co-leadership; (4) clarity about roles and responsibilities of partners; (5) recognition of benefits to both parties and alignment of the project’s aims with interests of each party; (6) sufficient funding to compensate for time and resources of both parties; and (7) use of community liaisons and conveners. 

Emerging health issues require innovative and creative solutions which necessitate community participation and engagement. For example, the COVID-19 pandemic generated many academic–community coalitions (e.g., [32,33,34]). The CBPR approach is effective at identifying and addressing social determinants of poverty, discrimination, and racism [15]. Its guiding principles support community members as equal partners in all research stages toward collaborative knowledge creation and action [35], linking diverse stakeholders including academic researchers, community organizations, and public health practitioners to work together. In the last decade, it has been established as a valued research approach within health education, public health, and other health and social science disciplines for its effectiveness in reducing inequalities [15,36]. A community–academic partner-based, integrated, and applied program can be effective for professional development and establishing innovative partnership between academics and practitioners [31]. To advance health equity, it is essential to work with community advocates and community-based organizations as they best understand the current status and needs of the affected communities [37]. 

It is our hope that the findings from the current research suggest insights into how to create effective collaborative partnerships and group dynamics to better serve communities in the future. 

## Figures and Tables

**Table 1:** Challenges and lessons learned from our experience of CAP.

	Challenges	Lessons Learned
Environment	Mutual understanding and team building take time and resources. The time and resources the community organization has to spend to establish a partnership strained its limited resources (e.g., limited financial resources, understaffing, etc.).	Without any pre-existing history of partnership, community–academic team building can be facilitated by community liaisons. Community–academic team building process can be benefited from team building funding.
Membership Characteristics	During the early stage of CAP, it can be unclear whom one can contact for questions that arise from the project. Community organizations and academic researchers have different needs, goals, cultures, and ways of working in their job, and the differences pose challenges to communication. Unclear expectations regarding the roles and responsibilities of both parties can work as a hindrance.	CAP can benefit from having a representative from both the community organization and academic research team to be in constant loop of communication. Members of community organizations and academic researchers should have mutual respect and understanding for their time, obligations, and responsibilities.
Process and Structure	CAP involves sustained efforts to purposefully involve both community partners and academic researchers in all phases of the research. Nurturing a co-led collaborative structure involves flexibility from both parties.	Involving both parties equally in any decision-making process enables the formation of a shared sense of vision, co-ownership, and co-leadership. Being flexible with the timing of meetings is essential to ensure everyone’s participation in the process of collaboration.
Communication	The early stage of CAP involves uncertainties regarding the proper mode, frequency, scope, and turnaround time of communication. Each party has its own priorities, and thus, preferred amount, mode, and frequency of communication can differ.	Community–academic team should consider having a conversation on the preferred means of communication at the beginning and establishing an open and transparent communication channel. Community–academic team should set the expectations as to the typical turnaround time.
Purpose	A shared understanding of the project’s purpose and goals is essential for a successful CAP. Yet, such shared understanding can be abstract and vague at the beginning.	Concrete outcomes from the project helped both parties to see the values of collaboration and partnership, bolstering the shared understanding of the project’s purposes and goals. A document with a set of goals and tangible outcomes along with clear timelines can help both parties to envision the project’s progress and motivate both parties.
Resource	Limited or no funding to compensate for the resource and efforts spent on the project makes the commitment to the project seen as excessive.	As CAP requires resources from both ends, the project should include a budget for compensating the time and effort of both parties. Conveners can be useful for collaboration among large-sized team members.
